# Exploring the mycobiota in multiple sclerosis: its influence on disease development and progression

**DOI:** 10.3389/fimmu.2025.1625794

**Published:** 2025-07-23

**Authors:** Ane Otaegui-Chivite, Miriam Gorostidi-Aicua, Laura Martins-Almeida, Ainhoa Alberro, Leire Romarate, Idoia Mendiburu, Amaya Álvarez de Arcaya, Maialen Arruti, Tamara Castillo-Triviño, David Otaegui, Laura Moles

**Affiliations:** ^1^ Group of Neuroimmunilogy, Biogipuzkoa Health Research Institute, San Sebastián, Spain; ^2^ Center for Biomedical Research Network in Neurodegenerative Diseases (CIBER-CIBERNED-ISCIII), Madrid, Spain; ^3^ Neurology Department, Hospital Universitario Donostia, Osakidetza Basque Health Service, San Sebastián, Spain; ^4^ Neurology Department, Hospital Universitario Araba, Osakidetza Basque Health Service, Vitoria, Spain

**Keywords:** mycobiome, multiple sclerosis, chitotriosidase, calprotectin, HLA-DRB1*1501

## Abstract

**Background:**

Multiple sclerosis (MS) is a complex immune-mediated disorder influenced by genetic, environmental, and microbial factors. Recent research has focused on the gut microbiota’s role in MS, yet limited studies have examined the fungal microbiota (mycobiota) in this context.

**Methods:**

In this study, we characterized the gut mycobiota of individuals with MS from the Basque Country, identifying specific fungal taxa associated with MS risk factors, clinical severity, and dietary patterns.

**Results:**

Our results revealed higher fungal diversity and richness in MS patients compared to controls, with significant enrichment of certain genera, including *Saccharomyces*, *Torulaspora*, and *Malassezia*. *Malassezia* demonstrated a strong association with increased disability, aligning with its previous identification in neurodegenerative conditions. Furthermore, we found that the presence of the MS-associated allele HLA-DRB1*1501 significantly influenced mycobiota composition and correlated with disability metrics. Additionally, we observed a complex interplay between plasma biomarkers (chitotriosidase and calprotectin) and specific fungal groups, with disease-specific correlations indicating potential interactions between the immune response and gut fungi. Notably, dietary fats showed a greater impact on mycobiota composition in MS patients than in controls, highlighting altered lipid metabolism in MS.

**Conclusion:**

These findings provide new insights into the fungal component of the gut microbiota in MS and underscore its potential role in disease pathogenesis and progression. Our work suggests that fungal biomarkers, together with genetic and dietary factors, may help refine our understanding of MS and support the development of mycobiota-targeted therapies.

## 1 Introduction

Multiple Sclerosis (MS) is a complex, chronic and immune-mediated disease characterized by inflammation, demyelination and progressive neurodegeneration. It arises from an immune response against self-antigens in individuals with a genetic predisposition (1). MS primarily affects young adults, with women being three times more likely to develop the disease than men (2). While the etiology of MS remain unclear, genetic factors are believed to account for approximately one-third of the disease risk (3). Environmental factors and lifestyle choices are also recognized as additional contributors on MS susceptibility. In the last decade, significant emphasis has been placed on studying the gut microbiota in the context of autoimmunity and neurodegeneration. Several published research studies report an altered gut microbiota in individuals with MS (4, 5), indicating a role in the pathogenesis of the disease. These studies evaluate the influence of the gut microbiota on immune responses and its potential contribution to the development and progression of MS. In fact, changes in microbial populations are thought to affect the function of the immune system, the formation of chronic inflammation and the integrity of barriers, all of them implicated in the MS pathogenesis (6). While most studies today are focused on the bacterial component of the gut microbiome, relatively little is known about another fundamental part, the mycobiota (the fungal community in the gut), with potential to influence on both, disease progression and immune function. Many links between fungi and diseases involving chronic inflammation have been found recently. Dysbiosis of the fungal communities has been observed in inflammatory bowel syndrome (IBD) (7), with studies highlighting the impact of these alterations on the inflammatory response (8) and the identification of potential fungal markers (9). In the context of MS, early indications of fungal involvement were based on findings of elevated serological antibody titers specific to *Candida* in individuals with MS compared to controls (10, 11). Besides, the presence of specific enzyme activity of *Candida albicans* correlate with disease severity (12). DNA from different fungal species has been detected in the CNS of individuals with MS (13, 14), suggesting a fungal infection in cerebral tissue. Moreover, systemic infections by non-*albicans Candida* strains have been shown to influence both the immunological and clinical aspects of experimental autoimmune encephalomyelitis (EAE), a model of MS, further suggesting their possible relevance in MS development (15, 16).

Previous studies describe the human mycobiota as dynamic and variable between individuals. At least 75 genera and 267 species have been identified in the human gut (17, 18). Most studies agree on the dominance of the phyla Ascomycota and Basidiomycota, with smaller proportions of Mucoromycota and Zoopagomycota. Among these, three genera, *Candida*, *Saccharomyces* and *Aspergillus*, are considered the most prominent (10, 17, 19). However, like the microbiota, there is no consensus on what constitute a “healthy” mycobiota (17). Despite this lack of consensus, numerous publications associate dysbiosis of the intestinal mycobiota with various diseases, particularly those linked to chronic inflammation and neurological disorders, such as obesity, IBD, autism, schizophrenia, and Alzheimer’s disease (20–23).

The role of infections in MS remains a subject of active debated, as definite proof of autoimmunity is still lacking. Various pathogens have been associated with the development or exacerbation of MS, including bacteria such as *Chlamydia pneumonia* and endotoxins produced by *Staphylococcus aureus*, which act as superantigens. Viruses from the *Herpesviridae* family, including Epstein–Barr virus (EBV) and human herpes virus 6 (HHV-6), as well as human endogenous retroviruses, have also been implicated (24). Among them, EBV stands out as having the strongest link to MS. EBV infects approximately 90% of the general population during the first decade of life and remains in the body as a latent infection, primarily within memory B cells. This persistent presence has been closely tied to the development and progression of MS, although the exact mechanisms linking the virus to MS pathology remain under investigation. Leading hypothesis include the reactivation of EBV within memory B cells in the CNS, the cross-reactivity of anti-EBV antibodies with human proteins in the CNS and the activation of “forbidden” memory B cells that recognize CNS antigens (25). However, despite the strong link between EBV and MS, no single pathogen has been definitely accepted as causal agent of the disease. Fungal infections, though less explored, have also been cautiously examined in the literature as potential contributors to MS pathogenesis. Some studies have shown symptoms improvement with antifungal treatments and the presence of antibodies against fungi in both serum and cerebrospinal fluid (CSF) of MS patients (16). Moreover, several known MS risk factors are linked to an increased susceptibility to fungal infections. Fungal populations in the human gut, similar to bacterial communities, are closely correlated with age. However, fungi also exhibit notable differences based on sex. The primary sex-based distinction is the higher prevalence of the genus *Candida* in the gastrointestinal tract of women (22). Since *Candida* is a commensal microorganism of the female genitourinary tract, it is thought to serve as a “natural” reservoir for the gastrointestinal tract. Vertical transmission (mother to child) of *Candida* has been documented, but *Candida* is generally not detected in the gut until adolescence (26, 27), a period during which the first symptoms of MS often emerge (usually between the ages of 20 and 30). These observations suggest a potential connection between *Candida* colonization and the increased risk of MS, although further research is needed to clarify this connection.

The HLA-DRB1*15 allele group is the strongest genetic risk factor for MS (28). This gene variant plays a critical role in immune system regulation and antigen presentation, determining which antigens trigger a T cell-mediated immune response and supports the maturation of naive B cell. The specific alleles HLA-DRB1*1501 and HLA-DRB1*1503, which confer the greatest predisposition to MS, have also been linked to an increased incidence of infections suspected to have a fungal etiology, such as pulmonary sarcoidosis, uveitis, or allergic bronchopulmonary aspergillosis (16).

The potential fungal etiology of MS is also being explored based on the beneficial effect of dimethyl fumarate (DMF), an immunomodulatory drug for MS. The proposed mechanisms by which DMF modulates the immune response include its anti-inflammatory interaction with the nuclear factor (erythroid-derived 2)-like 2 (Nrf2) pathway. The efficacy of DMF has been demonstrated by its ability to reduce relapse rates by about 50% as compared with placebo and reduce the disability progression. However, the exact mode of action remains incompletely understood (29). Notably, DMF has long been used as an industrial fungicide to suppress mold growth, suggesting that its efficacy in alleviating MS symptoms may also stem from a direct antifungal effect (16).

Additionally, the presence of chitotriosidase in CSF is an important biomarker of MS. Chitotriosidase is produced by activated macrophages to hydrolyze chitin, a polysaccharide found in fungi and absent in prokaryotic or mammalian cells. The reason for elevated chitotriosidase in MS patients’ CSF is not fully understood, but it may be linked to the presence of fungi or a microbiota rich in fungal organisms (30, 31). Calprotectin is another key biomarker particularly elevated in the CSF of MS patients during disease relapses. Located in the cytosol of neutrophils, calprotectin is an antimicrobial protein with strongly activity against fungi, further supporting the hypothesis of a fungal involvement in MS (16).

This study seeks to investigate the composition of the gut mycobiota in MS patients across different treatment conditions and disease stages, compared to healthy controls. By integrating microbiota analysis with inflammatory markers such as calprotectin and chitotriosidase, as well as genetic data on the HLA-DRB1*15 alleles, we aim to uncover potential links between gut fungal communities, immune function, and genetic risk factors in MS. These findings may lead to the identification of new therapeutic targets and provide insights into how treatments like DMF affect both the gut mycobiome and MS progression.

## 2 Materials and methods

### 2.1 Participants and sample collection

We conducted a prospective observational case-control study involving 50 individuals diagnosed with Multiple Sclerosis (MS) from Hospital Universitario Donostia (Donostia-San Sebastian, Spain) and Hospital Universitario Araba (Vitoria-Gasteiz, Spain), between December 2021 and June 2023, alongside 25 healthy controls (HCs) of the same age range. The local ethics committee approved the research protocol (reference PI2020070) on July 14, 2021. Written informed consent was obtained from all participants.

To qualify for enrolment, MS participants needed confirmed MS diagnosis according to the revised McDonald criteria (32). Exclusion criteria included gastrointestinal or chronic infectious diseases, recent steroids use (within the last month), chemotherapy or antibiotics treatment (within the last three months), pregnancy, or being within six months postpartum. Additionally, individuals with MS must have been on the same disease modifying therapy (DMT) for at least the last three months. MS participants were either treatment-naïve or treated with Dimethyl fumarate (Tecfidera^®^). HCs were required to have no autoimmune, gastrointestinal or chronic infectious diseases and not be relative to MS subjects.

Participants were provided with materials and instructions for fecal sample collection, including a stool collector designed to adhere to the toilet, tubes to collect stool samples, a safety bag, and hydrated cold packs to maintain the required temperature. Upon submission of frozen fecal sample, blood was drawn via venipuncture for plasma and DNA extraction. Samples were store at -80°C until analysis. At recruitment, participants completed a food frequency questionnaire to assess their eating habits, and test included in the Multiple Sclerosis Functional Composite (MSFC) were performed to measure various levels of disability in patients. Relevant clinical and demographic data, along with key questionnaire results, are summarized in **Table 1**.

**Table 1:** Clinical and demographic data of the studied population and summary of their dietary habits.

Sample type n (%)	Ctl 25 (33.3)		Naive MS 24 (32.0)				DMF MS 26 (34.7)				p. value
Age (years) median (IQR)	48.0 (42.0; 57.0)		55.5 (45.3; 64.0)				44.5 (38.0; 49.5)				0.007
Sex n (%)	Woman	Man	Woman		Man		Woman		Man		0.001^
	5 (25)	20 (75)	15 (62.5)		9 (37.5)		17 (65.4)		9 (34.6)		
Presence of mycobiota n (%)	Yes	No	Yes		No		Yes		No		0.008^
	20 (75)	5 (25)	18 (79.2)		5 (20.8)		11 (42.3)		15 (57.7)		
Clinical condition											
EDSS score n (%)	–	–	0	<3		≥3	0	<3		≥3	0.046^
			9 (37.5)	8 (33.3)		7 (29.2)	7 (26.9)	17 (65.4)		2 (7.7)	
Years of evolution median (IQR)	–	–	12.0 (8.0; 17.0)				8.5 (6.0; 15.0)				0.136
MSSS median (IQR)	–		1.0 (0.2; 2.9)				1.0 (0.3; 2.9)				0.936
MSFC median (IQR)	–		-0.0 (-0.3; 0.3)				-0.1 (-0.3; 0.1)				0.469
Dietary habits											
Protein (%kcal) median (IQR)	23.3 (21.2; 31.1)		26.0 (21.0; 36.7)				26.5 (14.6; 39.4)				0.796
Total Fat (%Kcal) median (IQR)	33.2 (28.8; 42.0)		40.4 (34.0; 59.1)				40.2 (29.8; 56.2)				0.278
SF (%Kcal) median (IQR)	8.5 (7.6; 9.4)		9.4 (7.5; 14.0)				10.6 (7.4; 12.8)				0.443
MUFA (%Kcal) median (IQR)	16.7 (13.6; 19.8)		17.4 (15.9; 23.9)				15.9 (12.8; 21.8)				0.367
PUFA (%Kcal) median (IQR)	3.8 (3.1; 4.7)		5.0 (3.4; 7.0)				4.4 (3.6; 6.2)				0.093
Carbohydrates (%Kcal) median (IQR)	45.3 (33.8; 52.1)		44.6 (36.8; 67.9)				47.2 (29.6; 58.5)				0.692
Fiber (g) median (IQR)	28.4 (23.7; 42.9)		41.7 (32.8; 49.2)				35.7 (16.9; 44.1)				0.228
Cholesterol (mg) median (IQR)	191.7 (140.1; 218.9)		278.0 (164.8; 366.1)				309.9 (172.5; 405.6)				0.029

Kruskal Wallis test.

^Fisher exact test.

### 2.2 DNA extraction and sequencing

Fecal samples were thawed on ice and diluted in 1X Dulbecco’s Phosphate Buffered Saline (DPBS) (Gibco, USA). DNA extraction was performed using both mechanical and enzymatic lysis, and the extraction kit QIAamp DNA Stool Mini Kit (Qiagen, Germany). Mechanical disruption involved bead-beating with 0.1 mm diameter zirconia/silica beads (Sigma, St.Louis, USA) using the Bead Ruptor 12 (OMNI International, Georgia, USA), conducted 1 minute three times. Enzymatic lysis of fungal cells was achieved with 0,1mg/ml zymolyase (MP Biomedicals, LLC, Illkirch-Graffenstaden, France). The extracted DNA underwent quality and quantity assessments. A negative control, extracted in parallel using an empty sterile tube (similar to those used for fecal samples) without any biological material, was included to evaluate the impact of reagents and potential contamination.

Amplification of the fungal ITS1 and ITS2 intergenic regions was performed using the primers ITS1 30F (5′- GTCCCTGCCCTTTGTACACA -3′) and ITS1 217R (5′- TTTCGCTGCGTTCTTCATCG -3′); as well as ITS86F (5’- GTGAATCATCGAATCTTTGAA -3’) and ITS4R (5’- TCCTCCGCTTATTGATATGC -3’), with AmpliTaq Gold™ DNA Polymerase (Applied Biosystems). The thermal cycling conditions were 95°C for 10 minutes, followed by 35 cycles of 95°C for 30 seconds, 55°C for 30 seconds, and 72°C for 1 minute. The PCR products from each fecal sample were pooled and sequence on Ion Torrent PGM (Life Technologies, MA, USA) using a 318 chip.

### 2.3 Data processing

ITS analysis was conducted using the QIIME2 microbiome bioinformatics platform. The pipeline performed in QIIME2 included the following steps: data import, quality filtering and denoising (using cutadapt to remove primers), ASV identification (via DADA2 denoise-pyro), diversity analysis (with reads rarefied to 10,000) and taxonomic classification using the UNITE database (through VSEARCH feature-classifier). Given the limited availability of pipelines for analyzing Ion Torrent sequences, the optimal approach for this analysis has been a topic of discussion within the QIIME2 community forum. The specific steps described here were selected based on recommendations and discussions in the forum (33–36). The pipeline designed and used for the microbiota analysis has been deposited on GitHub, https://github.com/MGorostidi/mbiome. (Gorostidi-Aicua, M. (2024) Mbiome. GitHub repository. https://github.com/MGorostidi/mbiome).

### 2.4 HLA-DRB1*15 allele genotyping

DNA was extracted from the buffy coat by the Basque Biobank (biobancovasco.org) using the FlexiGene DNA Kit (QIAGEN) and stored at -20˚C until use. Samples were thawed on ice prior to analysis.

Genotyping was performed using the TaqMan^®^ SNP Genotyping Assay (Thermo Fisher). This assay employed a primer pair and two allele-specific probes, labeled FAM and VIC, to detect G and A nucleotides of the SNP rs3135388, respectively. Quantitative polymerase chain reaction (qPCR) was conducted using the TaqPath ProAmp Master Mix and 20X TaqMan SNP Genotyping Assay with 10ng of genomic DNA per well on 384-well plates. Reactions were carried out in a total volume of 5 µl using the CFX384 instrument (BioRad). The qPCR protocol included a pre-read step at 60°C for 30 seconds, an initial denaturalization at 95°C for 5 minutes, followed by 40 cycles of 95°C for 15 seconds and 60°C for 1 minute. Fluorescence detection occurred at 60°C. Control samples representing all possible genotypes and a negative template control (NTC) were included in each run.

### 2.5 Calprotectin and chitotriosidase ELISAs

Calprotectin concentration in plasma was measured with Human Calprotectin L1/S100-A8/A9 Complex ELISA Kit (Catalog# EH62RB, Invitrogen) following the manufacturer’s instructions. Plasma samples were diluted 1:400 to fit the standard curve of the kit. Plasma chitotriosidase levels were measured using the Human Chitotriosidase ELISA Kit (Catalog# EH105RB, Invitrogen) according to the manufacturer’s instructions. Plasma samples were diluted 1:80 to fit the standard curve provided by the kit.

### 2.6 Food Frequency Questionnaire analysis

The FFQ included 142 food items, with intake recorded in the following categories: never, hardly ever, monthly (1–3 times per month), weekly (1–6 times per week) and daily (1 to more than 6 times per day). The food items were based on the validated FFQ used in the PREDIMED study, with slight modifications (37). The questionnaire covered a wide range of foods typically consumed in a Mediterranean diet. The nutritional value of each item was calculated using food composition tables from the Universidad Complutense of Madrid (38), which provides detailed nutritional information for 960 food items, expressed per 100 grams of edible portion.

The percentage of calories provided by each macronutrient was calculated by converting grams to calories (4 Kcal/g for proteins and carbohydrates, and 9 Kcal/g for fat) and then determining the percentage relative to the total caloric intake.

The estimation of adequate/inadequate intake of the main macro and micronutrients has been based on WHO recommendations (39).

### 2.7 MSFC determination

Multiple Sclerosis Functional Composite (MSFC) is a multidimensional, three-component performance scale used to assess the degree of impairment in MS patients. Timed 25-Foot Walk (T25W) for leg function and ambulation, 9-Hole Peg Test (9HPT) for arm and hand function, and Symbol Digit Modalities Test (SDMT) for cognitive function. These tests were explained to participants and performed at the time of signing the documentation or during sample delivery.

Since the units of three variables measured by these tests are different, raw scores are converted to common metric, z-scores. The overall composite score is calculated as the average of the z-score from the three tests. Negative scores represent those patients with an evolution better than average and positive values indicate worse evolution.

### 2.8 Statistical analysis

Statistical analysis was done with R software version 4.3.1 (http://cran.r-project.org). Quantitative variables were expressed as mean and 95% coefficient interval. Categorical variables were expressed as numbers and percentages. Kruskal-Wallis test was used to compare between individuals with MS and controls in the quantitative variables, while Two Tailed Fisher exact test was used to compare groups in categorical variables. Pearson’s correlation was used to describe the association between the quantitative variables. All plots were performed in R environment. Correlation matrices were achieved by using the R corrplot package (v0.94) and significant correlations were calculated using Hmisc package (v5.1.3). ROC curves were performed with pROC R package (v1.18.5). AUC and cut point were calculated with the same package. PCA was performed using r packages ggfortify (v0.4.17) and ggplot2 (v3.5.1). P-values ≤ 0.05 were considered statistically significant.

## 3 Results

A total of 50 individuals with MS and 25 controls participated in the study. Participant ages ranged from 27 to 87 years across groups. The MS subjects treated with DMF were generally younger, with a median age of 44.5 years and older in naïve individuals (55.5 years as a median) versus controls (median age of 48 years) (p = 0.007). Following the trends in the incidence of the disease, women were more frequently represented in the MS group, while men were more common in the control group (p = 0.001).

MS treatment with DMF was associated with a significant reduction in the gut mycobiota presence; dropping from 79.2% in naïve group to 42.3% in DMF treated individuals. Mycobiota was present in 75% of controls (p = 0.008).

Disability was assessed through EDSS scores, categorized as 0, <3 and ≥3. Slight differences in EDSS distribution were observed within MS subjects, being the intermediate form by far the predominant one in individuals treated with DMF (p = 0.046). However, no significant group differences were found for the Multiple Sclerosis Severity Score (MSSS) (p = 0.936) or the MS functional composite (MSFC) score (p = 0.469) (**Table 1**).

No differences were observed in the dietary habits of participants, except for cholesterol intake, which was higher among individuals with MS (p = 0.029).

### 3.1 Differential diet-fungus correlation pattern in individuals with MS suggests disease-specific macronutrient metabolism

**Figure 1:**
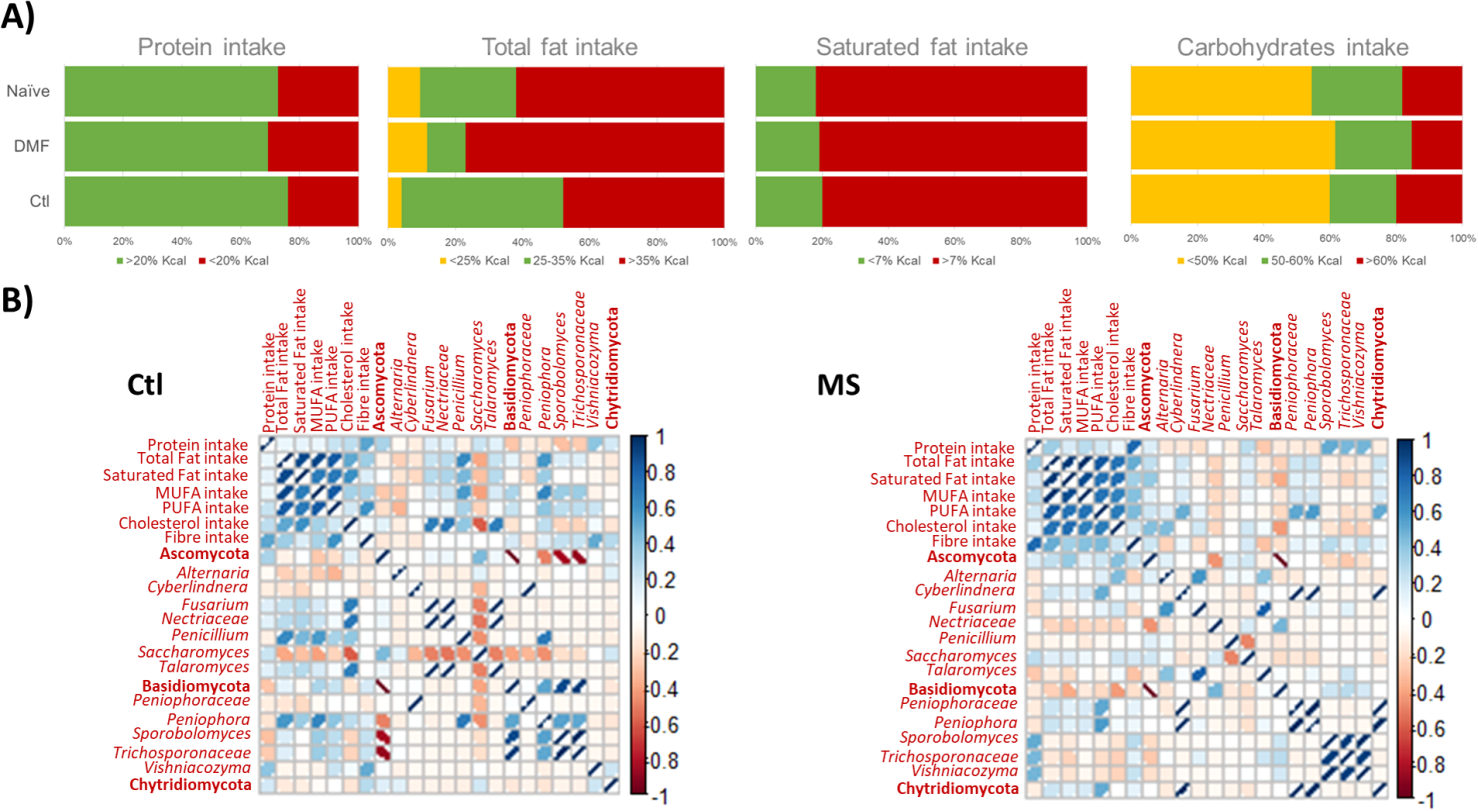
**(A)** Barr plot illustrating the intake of primary macronutrients among study participants, categorized by MS status and type of disease modifying therapy. Intake levels were evaluated against WHO recommend guidelines. **(B)** Pearson's correlation matrix depicting significant correlations between dietary components and fungal populations, with comparisons between the control (Ctl) and MS (MS) groups. Positive correlations are represented in blue, and negative correlations in red; the intensity of color and the width of ellipses correspond to the strength of the correlation.

The analysis of FFQs indicates that the dietary habits of individuals with MS are generally similar to those reported by controls, with the differences primarily limited to higher cholesterol consumption among those with MS (p= 0.029). Protein intake was within recommended levels for most participants, while total fat consumption, particularly of saturated fats, was excessive, and carbohydrates intake fell short of recommended values for the majority (**Figure 1A**).

Correlation patterns between major macronutrients and fungal populations differed between controls and individuals with MS (**Figure 1B**). Specifically, cholesterol intake was associated with levels of main fungal phyla, Ascomycota and Basidiomycota, in the MS group but not in controls. At more specific taxonomic levels, cholesterol intake was linked to increased levels of *Talaromyces* (p = 0.010), *Nectriaceae* (p = 0.006) and *Fusarium* (p = 0.010) in controls, and to *Alternaria* (p = 0.040) in MS patients. In controls, *Saccharomyces* levels decreased with higher intake of cholesterol (p = 0.060). The genus *Peniophora* correlated with total fat and MUFA intake in controls (p = 0.002) and with PUFA intake in individuals with MS (p = 0.005). *Penicillium* was correlated with total fats (p = 0.003), saturated fats (p = 0.046) and MUFA intake (p = 0.010) in controls, but no significant correlation was observed in MS patients. Additionally, *Cyberlindnera* was associated with PUFA ingestion in individuals with MS (p = 0.016) but not in controls. A positive correlation was observed between *Vishniacozyma* and fiber intake in controls (p = 0.024), whereas in MS patients, *Vishniacozyma* (p = 0.025), *Sporobolomyces* (p = 0.013) and *Trichosporonaceae* (p = 0.030) were associated with protein intake (**Figure 1B**).

### 3.2 The mycobiome of individuals with MS exhibits disease specific characteristics and is partially influenced by DMF treatment

The total DNA content per gram of feces ranged from 6.12 and 760.28 ng, with a mean of 133.24 ng/g, while no DNA was detected in the negative control. DNA quantity was standardized prior to amplification and again before sequencing.

The average total reads in the samples was 47598, with the number of genera ranging from 2 to 43. Negative controls exhibit similar total reads and Shannon diversity index to the samples; however, their Ascomycota/Basidiomycota ratio and taxonomic profile were distinctly different from those observed in the samples (**Supplementary Figure 1**). Most of the reads predominant in the negative control samples (except for *Saccharomyces*) were nearly undetectable in the samples (<0.5%). Therefore, we considered contamination background and ubiquitous DNA in reagents to be unlikely to affect the mycobiota composition in fecal samples.

**Figure 2:**
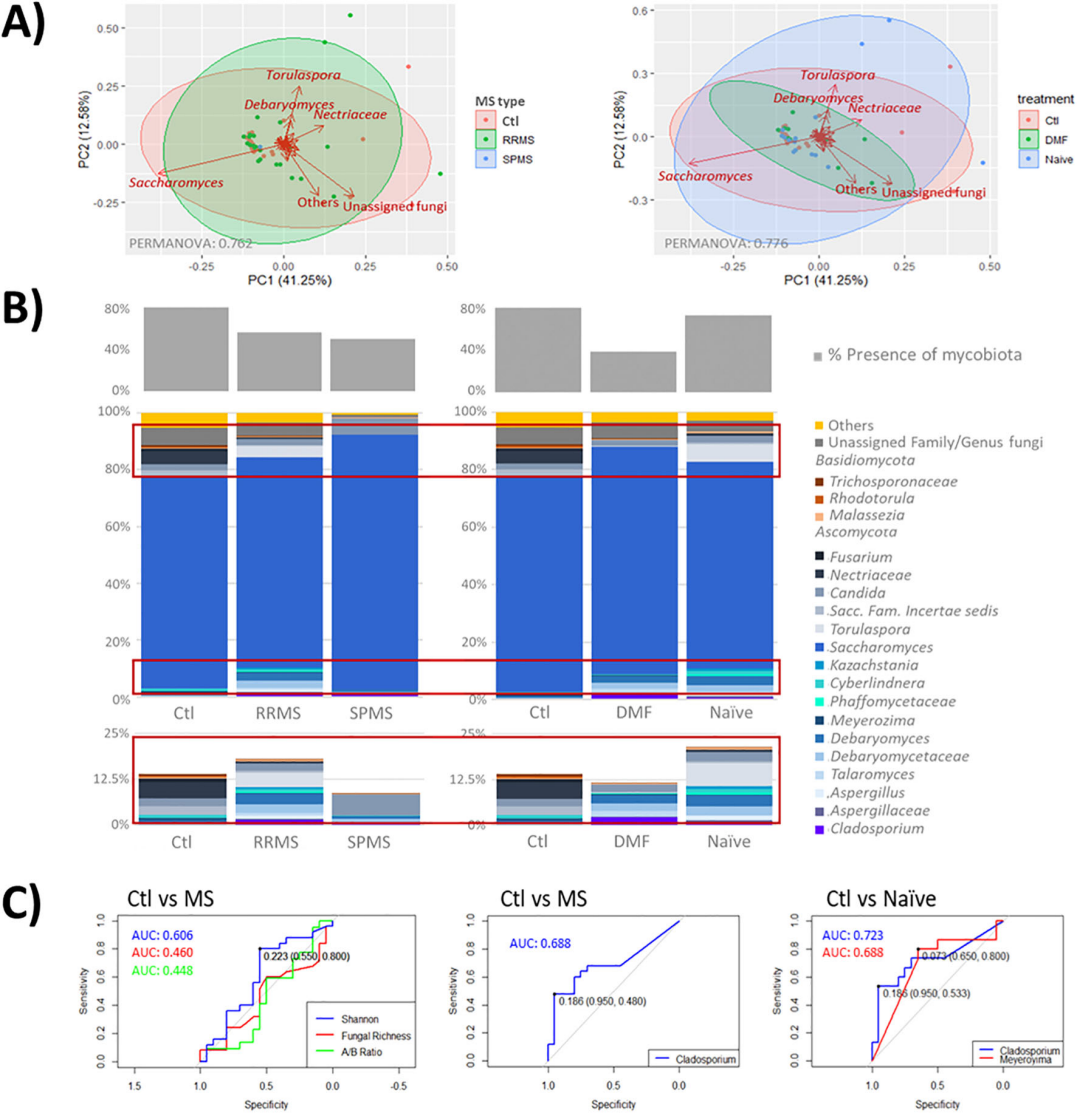
**(A)** Scatter plot representing samples grouped by disease type (left) and treatment status (right). Clustered samples were color-coded by group, with fungal groups that have the greatest influence on sample placement indicated by arrows. The bidimensional PCoA plot explains 53.83% of the variability among the samples. **(B)** Taxonomic analysis of mycobiota across samples and groups. The middle graph shows a taxonomy bar plot of the main phyla and genera (those accounting for >0.5%abundance and/or present in at least 10% of the samples). The upper graph illustrates the presence of mycobiota across the samples; with minority genera highlighted in red boxes are enlarged at the bottom. Note, where genus level resolution was not available, taxa are reported at the family level. **(C)** ROC curves illustrating the diagnostic performance of proposed mycobiome biomarkers (*Cladosporium* and *Meyerozyma*), with associated AUC values, compared to global parameters such as diversity (Shannon), fungal richness and the Ascomycota/Basidiomycota ratio.

The mycobiota composition did not show a clear clustering of the study groups. Nevertheless, the fungal genera *Torulaspora* and *Debaryomyces* were primarily associated with the MS mycobiota, particularly in treatment naïve participants, while *Saccharomyces* was characteristic of the control group. No distinct fungal taxa were identified in the mycobiota of patients treated with DMF (**Figure 2A**). Mycobiome detection was more frequent in controls than in people with MS, and DMF treatment determined a reduction in mycobiota presence. The Ascomycota phylum and the genus *Saccharomyces* predominated in the human mycobiota, with relative abundances ranging from 54.3% and 100% for Ascomycota and 0.01% and 99.96% for *Saccharomyces* across the samples (**Figure 2B**). A trend toward increased fungal diversity and richness was observed in people with MS, although no significant differences were found in the Ascomycota/Basidiomycota ratio between groups.

Key differences between individuals with MS and controls were primarily found in the minority taxa of the mycobiota (**Figure 2B**; **Table 2**). Specifically, reads from the genera *Cladosporium* (p = 0.022) and unassigned genera of the family *Sclerotiniaceae* (p = 0.039) were elevated in MS patients, while reads from *Meyerozima* (p = 0.043) and unassigned genera of the family *Saccharomycetaceae* (p = 0.039) were reduced. These differences were less pronounced in patients treated with DMF (**Table 2**).

**Table 2:** Fungal groups detected on the mycobiota of the participants in the study.

Microorganism	Ctl		DMF		NaÏve		p. value^1^	p. value^2^	p. value^3^
	n (%)	Median (IQR)	n (%)	Median (IQR)	n (%)	Median (IQR)			
*Cladosporium*	11 (55%)	0.01 (0.00-0.05)	6 (60%)	0.08 (0.00-0.35)	11 (69%)	0.19 (0.02-0.66)	0.215	0.022	0.685
*Meyerozyma*	13 (65%)	0.16 (0.00-0.19)	6 (60%)	0.15 (0.00-0.25)	4 (25%)	0.00 (0.00-0.34)	0.822	0.043	0.042
*Un. genera Saccharomycetaceae*	15 (75%)	0.00 (0.00-0.00)	8 (80%)	0.00 (0.00-0.00)	6 (38%)	0.00 (0.00-0.00)	0.157	0.039	0.041
*Un. genera Sclerotiniaceae*	0 (0%)	0.00 (0.00-0.00)	0 (0%)	0.00 (0.00-0.00)	3 (19%)	0.00 (0.00-0.00)	–	0.039	0.045
*Sporobolomyces*	0 (0%)	0.00 (0.00-0.00)	0 (0%)	0.00 (0.00-0.00)	3 (19%)	0.00 (0.00-0.00)	0.480	0.205	0.045

^1, 2^Kruskal-Wallis test.

^3^Fisher exact test.

^1^Comparison of patients treated with DMF versus Controls.

^2^Comparison of naïve patients versus Controls.

Un. genera Saccharomycetaceae, Unassigned genera of the family Saccharomycetaceae; Un. genera Sclerotiniacea, Unassigned genera of the family Sclerotiniacea.

Further analysis revealed that the presence of *Meyerozima* (p = 0.042) and unassigned genera of the family *Saccharomycetaceae* (p = 0.041) were less frequent in naïve MS patients, while the presence of unassigned genera of the family *Sclerotiniaceae* (p = 0.045) and the genus *Sporobolomyces* (p = 0.045) characterized the mycobiota of naïve patients (**Table 2**). Investigation of some of these genera as potential disease markers showed that *Cladosporium* abundance in the gut could predict MS with an AUC value of 0.688, while the combination of *Cladosporium* and *Meyerozima* levels differentiated between controls and naïve patients with AUC values of 0.723 and 0.688, respectively; higher than those obtained from general parameters (**Figure 2C**).

### 3.3 Mycobiota composition change with disease severity. Specific fungal population correlates with severity metrics of MS

**Figure 3:**
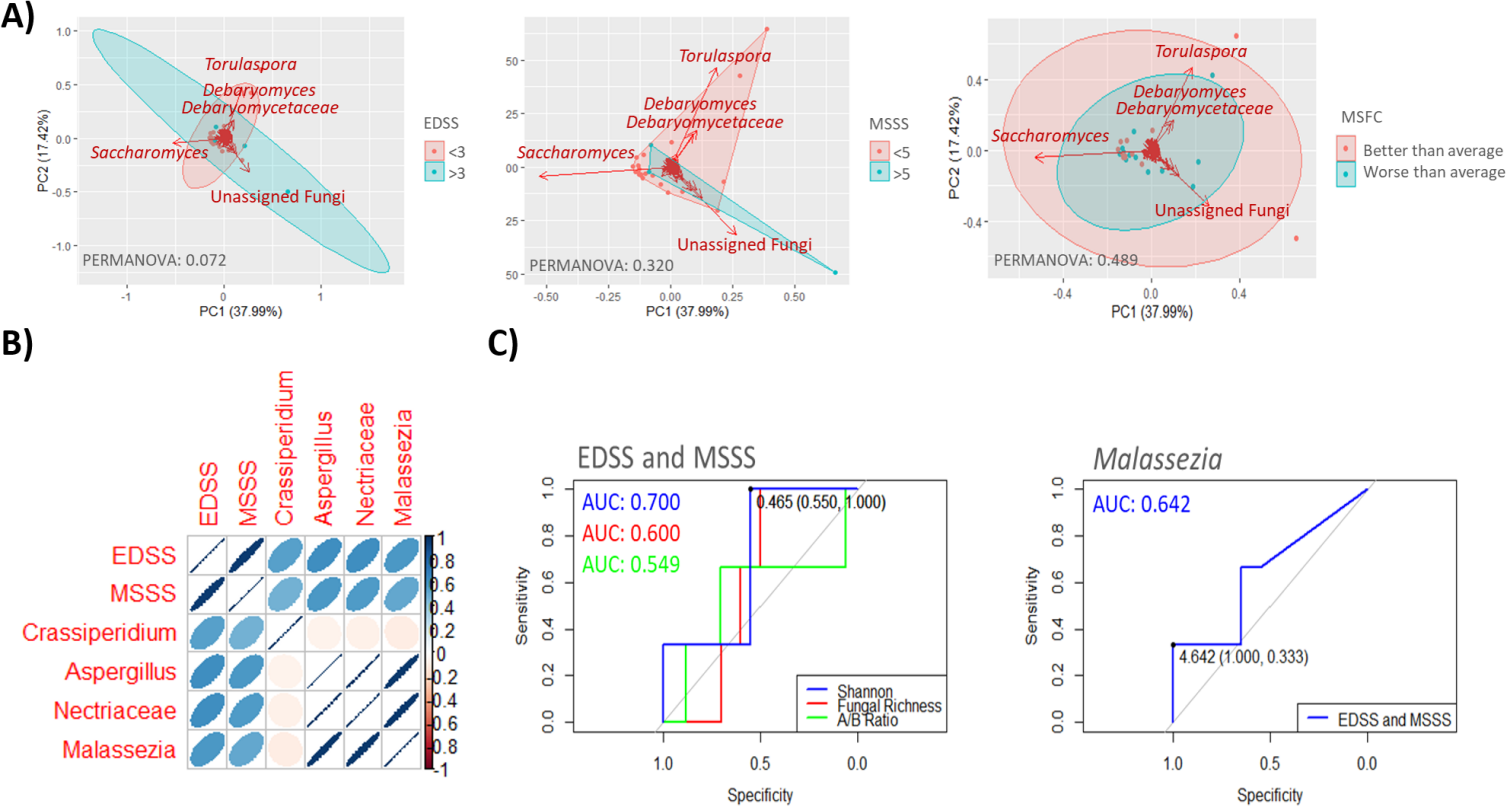
**(A)** Scatter plots showing sample groupings based on disease severity as determined by the EDSS score (≥3 or <3) (left), MSSS score (≥5 or <5) (middle) and MSFC score (better or worse than average) (right). Samples within clusters are shown in different colors, with arrows indicating the fungal groups most influential in determining sample placement. The bidimensional PCoA plot explains 55.41% of the sample variability. **(B)** Pearson correlation matrix depicting the relationship among four fungal genera significantly correlated (p < 0.05) with MS severity scales EDSS and MSSS. Color intensity reflects the correlation strength, with dark blue (1) indicating a complete positive correlation and dark red (−1) indicating a complete negative correlation. The shape of the ellipses denotes correlation strength, with narrower ellipses indicating stronger correlations and wider ellipses weaker correlation. **(C)** ROC curves illustrating the diagnostic performance of proposed mycobiome biomarkers, specifically *Malassezia*, in assessing severity as determined by EDSS or MSSS scores (right panel). Globally parameters such as diversity (Shannon), fungal richness and Ascomycota/Basidiomycota ratio are shown in the left panel.

All the severity metric used yielded similar results; *Torulaspora* was the fungal group most characterized of individuals with lower disability, while unassigned fungal genera predominated in those with higher disability levels. Although clustering was not statistically significant across any of the scales used, the EDSS scoring, categorized as <3 or ≥3, provided the most effective group separation (PERMANOVA: 0.072) (**Figure 3A**). Among specific microorganisms, those most strongly correlated with MS severity scores included *Crassiperidium* across all scales, as well as *Aspergillus*, *Nectriaceae* and *Malassezia* on the EDSS and MSSS scales (**Figure 3B**). *Malassezia* in the gut showed the highest predictive accuracy for MS severity on the EDSS and MSSS scales, with an AUC of 0.642. However, global mycobiome metrics, particularly alpha diversity measures, demonstrated even better predictive power, with Shannon Diversity index achieving an AUC of 0.700 (**Figure 3C**).

### 3.4 Mycobiota alteration and its relation with risk of MS susceptibility

**Figure 4:**
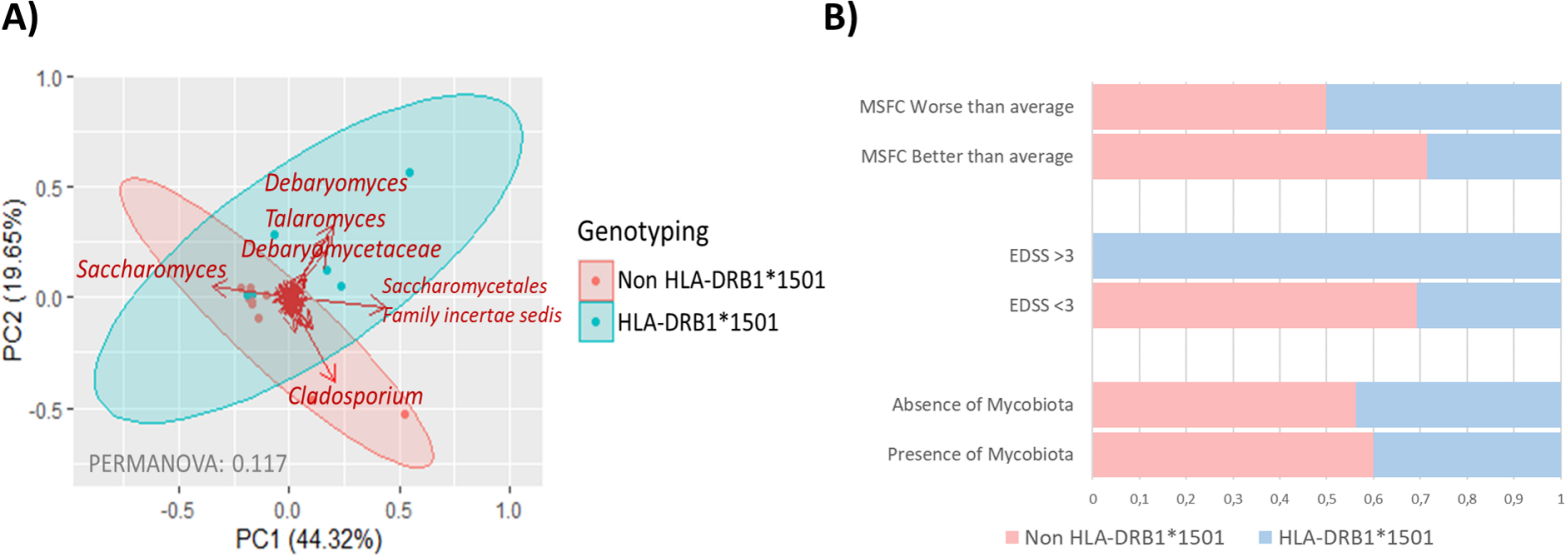
**(A)** Scatter plot illustrating sample groupings based on HLA-DRB1*15 allele genotyping, with carriers (HLA-DBR1*1501) represented in blue and non-carriers (HLA-DBR1*1501) in red. Arrows indicate the fungal groups most influential in determining samples placement. The bidimensional PCoA plot accounts for 63.97% of the variability in the samples. **(B)** Barr plot showing the distribution of HLA-DRB1*1501 allele carriers across MSFC and EDSS severity categories, alongside the presence of gut mycobiota in individuals with MS.

The mycobiota of individuals with MS demonstrated a significant correlation with the MS genetic risk factor HLA-DRB1*15 alleles. HLA genotyping revealed that individuals carrying the HLA-DRB1*1501 allele had a distinct mycobiota composition, characterized by the presence of *Debaryomyces* and *Talaromyces* genera. In contrast, the mycobiota of non-carriers was predominated by the genus *Cladosporium* (**Figure 4A**). Furthermore, carriers of the HLA-DRB1*1501 allele were more likely to exhibit more severe MS, as reflected by worse MSFC scores and higher EDSS scores. However, the overall composition of the gut mycobiota did not differ significantly between HLA-DRB1*1501 carriers and non-carriers (**Figure 4B**).

Plasma chitotriosidase levels did not differ significantly between individuals with MS and controls. Interestingly, in MS patients, chitotriosidase levels tended to increase with age (p = 0.027), a pattern not observed in controls. However, no significant correlation was observed between plasma chitotriosidase levels and MS severity metrics in the overall population, except for the higher levels in treatment naïve patients with worse prognosis, as indicated by a higher MSSS score (p = 0.028) (**Table 3**).

**Table 3:** Chitotriosidase plasma levels (ng/ml).

Status (n)	HC (25)		MS (50)		p. value	
	11.21 (9.80; 21.00)		12.54 (8.35; 21.00)		0.828	
Sex (n)	Woman (5)	Man (20)	Woman (32)	Man (18)	0.197^1^	0.317^2^
	21.00 (11.21; 28.88)	10.82 (9.34; 15.86)	12.11 (7.66; 20.57)	15.34 (10.01; 21.62)		
Age (years) (n)	<50 (16)	>50 (9)	<50 (28)	>50 (22)	0.865^1^	0.027^2^
	11.10 (9.40; 17.46)	11.96 (9.80;22.74)	9.49 (7.51; 15.16)	16.66 (11.17; 24.13)		
Presence of mycobiome (n)	Yes (20)	No (5)	Yes (26)	No (24)	0.197^1^	0.360^2^
	12.77 (9.35; 24.28)	10.29 (9.87; 11.21)	12.67 (9.31; 21.20)	11.98 (7.46; 16.86)		
MS Clinical conditions					p. value	
MS group (n)	Naïve MS (24)		DMF MS (26)		0.374	
	12.67 (8.71; 21.66)		11.98 (8.55; 16.60)			
EDSS (n)	<3 (17)	≥3 (7)	<3 (24)	≥3 (2)	0.575^3^	0.497^4^
	12.54 (8.78; 21.31)	19.08 (10.20; 23.46)	12.70 (8.34; 16.73)	8.97 (8.97; 8.97)		
MSSS (n)	<5 (20)	≥5 (4)	<5 (24)	≥5 (2)	0.028^3^	0.173^4^
	12.26 (7.88; 20.73)	24.04 (22.87; 30.19)	13.43 (8.97; 16.86)	8.22 (7.84; 8.59)		
MSFC (n)	Better than average (13)	Worse than average (9)	Better than average (9)	Worse than average (17)	0.065^3^	0.439^4^
	18.80 (12.67; 24.04)	9.76 (6.62; 20.73)	12.70 (10.86; 16.26)	10.00 (7.56; 16.60)		

Chitotriosidase levels are expressed as Median (IQR).

Kruskal-Wallis test.

^1^Comparison of Ctl groups.

^2^Comparison of MS groups.

^3^Comparison of Naïve MS groups.

^4^Comparison of DMF MS groups.

Plasma calprotectin levels do not show significant differences between individuals with MS and controls, nor were associated with age, sex, disease severity or treatment. However, a tendency for increased calprotectin levels was observed in men and in controls with the presence of gut mycobiota, a pattern not seen in individuals with MS (**Table 4**).

**Table 4:** Calprotectin plasma levels (ng/ml).

Status (n)	Ctl (25)		MS (50)		p. value	
	184.54 (100.53; 252.29)		110.26 (54.66; 189.12)		0.069	
Sex (n)	Woman (5)	Man (20)	Woman (32)	Man (18)	0.655^1^	0.734^2^
	121.65 (113.30; 213.82)	208.34 (97.82; 253.20)	110.26 (46.31; 168.80)	121.31 (72.87; 208.25)		
Age (years) (n)	<50 (16)	>50 (9)	<50 (28)	>50 (22)	0.333^1^	0.187^2^
	184.54 (117.47; 256.04)	170.91 (34.96; 244.95)	95.14 (42.62; 146.06)	132.33 (97.39; 236.46)		
Presence of mycobiome (n)	Yes (20)	No (5)	Yes (26)	No (24)	0.456^1^	0.408^2^
	199.18 (123.24; 253.20)	113.30 (47.67; 232.13)	107.23 (51.36; 142.50)	125.55 (69.23; 247.19)		
MS Clinical conditions					p. value	
MS group (n)	Naïve MS (22)		DMF MS (26)		0.846	
	109.64 (80.03; 160.34)		118.77 (36.07; 189.12)			
EDSS (n)	<3 (17)	≥3 (7)	<3 (24)	≥3 (2)	0.458^3^	0.821^4^
	121.35 (96.40; 183.66)	87.55 (63.42; 90.10)	113.50 (29.66; 193.23)	142.50 (142.50; 142.50)		
MSSS (n)	<5 (20)	≥5 (4)	<5 (24)	≥5 (2)	0.958^3^	0.300^4^
	110.26 (85.56; 137.03)	87.55 (75.48; 398.62)	118.77 (48.89; 197.35)	56.67 (13.75; 99.59)		
MSFC (n)	Better than average (13)	Worse than average (9)	Better than average (9)	Worse than average (17)	0.965^3^	0.747^4^
	109.02 (63.42; 133.60)	107.23 (85.56; 121.35)	142.50 (86.99; 185.00)	100.18 (32.81; 212.80)		

Calprotectin levels are expressed as Median (IQR).

Kruskal-Wallis test.

^1^Comparison of Ctl groups.

^2^Comparison of MS groups.

^3^Comparison of Naïve MS groups.

^4^Comparison of DMF MS groups.

**Figure 5:**
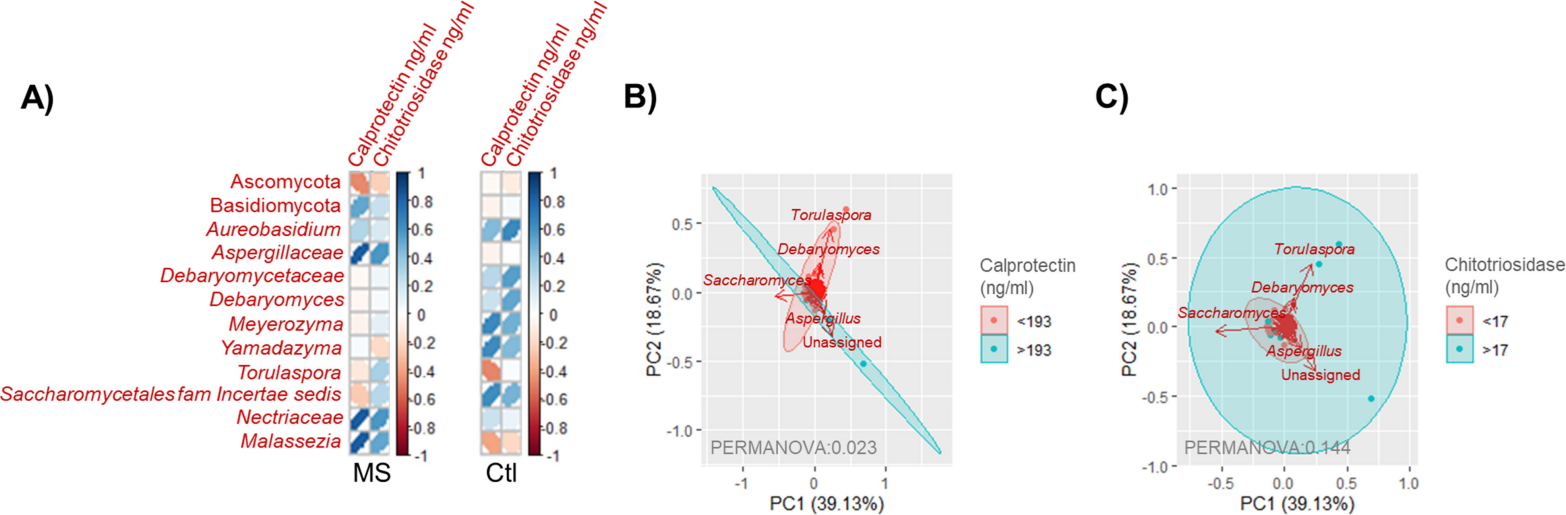
**(A)** Pearson correlation matrix showing fungal taxa significantly correlated (p < 0.05) with plasma calprotectin or chitotrisiosidase levels in individuals with MS or controls. Colors indicate the correlation strength, with dark blue representing a complete positive correlation (1) and dark red indicating a complete negative correlation (−1). The shape of the ellipses reflects correlation strength, with narrow ellipses indicating stronger correlations and wider ellipses weaker correlations. **(B)** Scatter plot representing samples of individuals with MS grouped by plasma calprotectin levels, categorized as <193 ≥193 ng/ml (the average concentration found in controls). Arrows indicate the fungal groups most influential in determining sample placement. The bidimensional PCoA plot explains 57.8% of the sample variability. **(C)** Scatter plot representing samples of individuals with MS grouped by plasma chitotriosidase levels, categorized as <17 ≥17 ng/ml (the average concentration found in controls). Arrows represent the fungal groups most influential in determining sample placement. The bidimensional PCoA plot explains 57.8% of the sample variability.

Several fungal groups showed significant correlations with plasma calprotectin and chitotrisidase levels, with these correlations being disease specific. Notably, higher calprotectin levels in MS patients were associated with a gut mycobiota composition characterized by reduced Ascomycota and increased Basidiomycota. Both plasma calprotectin and chitotriosidase levels displayed a positive correlation with the fungal families *Aspergillaceae* and *Nectriaceae*, as well as the genus *Malassezia* in individuals with MS but not in controls (**Figure 5A**). The mycobiota of MS patients with plasma calprotectin levels either higher or lower than the average observed in controls showed significant differences (permanova 0.023). Specifically, the genera *Saccharomyces* and *Aspergillus* were predominant in patients with higher calprotectin levels, while *Torulaspora* and *Debaryomyces* characterized the mycobiota of those with lower calprotectin levels (**Figure 5B**). In contrast, no differences were observed in the mycobiota of MS patients when stratified by plasma chitotriosidase levels (**Figure 5C**).

## 4 Discussion

Our previous studies have identified cross-kingdom interactions within the gut microbiota of individuals with MS from the Basque Country, including characterizing the bacterial gut microbiota in relation to disease modifying therapies for MS (40–42). In the current study, we expand our analysis by describing the fungal microbiota of this population and examining its associations with MS risk factors of different natures, as well as dietary habits.

The fungal microbiota of individuals with MS exhibited higher fungal diversity and richness, which is consistent with findings reported by other authors (43–45). While a general trend of increased Basidiomycota presence has been previously described in MS, we did not detect significant differences in the levels of Ascomycota or Basidiomycota in our cohort. However, we did observe a higher richness of the phylum Basidiomycota in individuals with MS compared to controls. The top five genera identified in our MS population were *Saccharomyces*, *Torulaspora*, *Debaryomyces*, *Candida* and *Cladosporium*. These findings align with previous studies, thought genera such as *Xylaria*, *Penicillium*, *Agaricus*, *Pichia*, *Aspergillus* and *Malassezia* have also been identified in other MS populations (43–45). Our analysis revealed that the main differences between individuals with MS and controls were found in minority genera, and no statistical differences in β-diversity parameters were observed.

We further explored the relationship between mycobiota composition and clinical metrics, finding several genera that positively correlated with EDSS and MSSS scores. Among these, *Malassezia* stood out as the most predictive genus for significant increases in disability, as measured by both EDSS (≥3) and MSSS (≥5) scores. This fungal genus, predominantly detected in women, has previously been linked to MS (46) and other neurodegenerative diseases (47–51). *Malassezia* is known for its affinity for lipids, and while it is typically a commensal of human skin and overgrowth on skin sebum, it has the potential to invade internal organ and cause infection, including the CNS, under certain conditions (50). The lipid rich environment of the brain (52), coupled with immune suppression and BBB disruption in MS, may facilitate the invasion of *Malassezia* into the CNS, potentially contributing to disease progression (47). Its presence has been identified on the CNS of individuals with Alzheimer (48) and MS (14).

Additionally, we found *Aspergillus*, a genus commonly associated with respiratory infections and known for mycotoxin production, also correlated with MS disability. Although CNS infections due to *Aspergillus* are rare, they can occur under specific conditions, especially in immunocompromised individuals (53, 54). The *Nectriaceae* family, which includes both saprophytic and pathogenic fungi, also correlated with disease severity, with the ubiquitous fungi *Fusarium* being of particular note. *Fusarium* has been documented as an opportunistic pathogen capable of invading the CNS, even in healthy individuals, though such infections are becoming more common, especially in immunocompromised patients (55).

Dietary habits in our study population revealed subtle differences between individuals with MS and controls. Notably, we observed distinct pattern of mycobiota composition associated with macronutrient intake, suggesting important metabolic differences in individuals with MS. In particular, cholesterol intake in controls was found to significantly influenced mycobiota composition, specifically in relation to fungal phyla abundances. Fats emerged as the most influential macronutrient in shaping mycobiota composition. Given the well-documented alterations in lipid metabolism in MS (56, 57), it is not surprising that dietary habits influence mycobiota composition differently in individuals with MS compared to controls.

We also explored the influence of MS genetic risk factors on mycobiota composition. The presence of the HLA-DRB1*1501 allele, a major genetic risk factor for MS (28), was found to significantly impact mycobiota composition, particularly in relation to MS related disability. The association between the HLA-DRB1*1501 allele and fungal infections has been widely discussed in the literature (58–60), and our findings further support its role in shaping both mycobiota composition and MS progression.

Our study also investigated the potential role of plasma chitotriosidase and calprotectin levels as biomarkers in MS. Previous research has report elevated levels of chitotriosidase in both the CNS (61) and plasma of MS patients (30). While we observed a slight increase in plasma chitotriosidase levels in MS patients, the differences were not statistically significant, though they were more pronounced in treatment naïve patients. These findings are consistent with those of Beliën et al., who linked increased chitotriosidase levels in CNS with microglia activation and proposed it as an early CSF biomarker for disability progression in MS (61). Interestingly, we observed marked gender and age related differences in chitotriosidase levels, particularly in MS patients. Although demographic parameters have not been extensively evaluated in relation to chitotriosidase levels, this warrant further investigation.

In contrast to studies reporting increased chitotriosidase levels with disease modifying treatments (e.g., β-interferons) (30), we found no significant effects of DMF, which has recognized antifungal activity, on chitotriosidase levels. This discrepancy may reflect the impact of DMF on both mycobiota composition and other immune related pathways. The correlation patterns between fungi, plasma chitotriosidase and calprotectin levels in individuals with MS and controls highlight the need for combined strategies to study microbiota composition, as the identification of potentially pathogenic species/strains may enhance our understanding of these differences.

Calprotectin, a calcium-binding protein involved in numerous biological processes, has been implicated in various inflammatory conditions, including MS. Previous studies have shown that calprotectin levels in CSF of MS patients are related to relapse rate (62) and plasma calprotectin levels are associated with active MS, as indicated by neurofilament light chain (NfL) levels (63). However, our study found no association between plasma calprotectin levels and disease status, disability, or progression. These results suggest that the influence of calprotectin may be more specific to CSF levels and disease activity rather than plasma levels. Interestingly, we did observe that plasma calprotectin levels influenced mycobiota composition (PERMANOVA: 0.023), aligning with previous findings suggesting a role for calprotectin in shaping the microbiota in inflammatory diseases (64).

In conclusion, this study highlights the distinct characteristics of the fungal microbiota in individuals with MS, emphasizing its potential role in disease severity and progression. The associations between specific fungal genera, such as *Malassezia* and *Aspergillus*, and clinical metrics like EDSS and MSSS scores, underscore the relevance of fungal dynamics in MS pathology. Moreover, the interplay between genetic factors like HLA-DRB1*1501, dietary influences, and plasma biomarkers such as chitotriosidase and calprotectin further illustrates the complexity of host-mycobiota interactions in MS. The work of the International Multiple Sclerosis Microbiome Study (iMSMS) consortium has firmly established an association between the bacterial component of the microbiota and MS. However, microbial communities do not exist in isolation, fungal and bacterial populations are closely interconnected, and changes in one community can lead to reciprocal shift in the other. These interactions are further modulated by host factors, creating a dynamic and multidirectional network of influences. Understanding MS pathogenesis thus requires moving beyond single-kingdom analysis and embracing a holistic view of the microbiota as an integrated ecosystem. This study not only highlights novel associations between the fungal microbiota and clinical, genetic, and inflammatory features of MS, but also sets the groundwork for the systematic characterization of minority microbial communities, such as fungi, within the broader microbiome landscape. By establishing and applying a targeted methodology for the analysis of these often-overlooked components, we demonstrate the feasibility and relevance of integrating fungal data into microbiome research.

These findings provide a foundation for future research to explore the functional implications of fungal dysbiosis in MS and its potential as a therapeutic target or biomarker. A multidisciplinary approach combining microbiota profiling, biomarker analysis and clinical insights is essential to fully unravel the role of the mycobiota in MS pathogenesis and management.

## Data Availability

The data presented in the study are deposited in the NCBI SRA repository, accession numbers from SAMN49790753 to SAMN49790798 and BioProject PRJNA1286116.

## Supplementary material

Supplementary Figure 1 Comparison of mycobiome profile in Negative controls (NC) and fecal samples. **(A)** Boxplot of the Ascomycota/Basidiomycota ratio. **(B)** Boxplot of the Shannon Diversity index. **(C)** Barr plot of the 10 more abundant taxa in the samples. Un. orders Dothideomycetes: Unassigned orders in the class Dothideomycetes; Un. genera *Debaryomycetaceae*: Unassigned genera in *Debaryomycetaceae* family; Un. genera *Nectriaceae*: Unassigned genera in *Nectriaceae* family; Un. families Pleosporales: Unassigned families in the order Pleosporales; Un. orders Sordariomycetes: Unassigned orders in the class Sordariomycetes; Un. classes Basidiomycota: Unassigned classes in the phyla Basidiomycota; Un. genera *Schizoporaceae*: Unassigned genera in *Schizoporaceae* family.
