# Supplementary figures and images for: Exploring the mycobiota in multiple sclerosis: its influence on disease development and progression
Source: Front Immunol. 2025 Jul 23;16:1625794. doi: 10.3389/fimmu.2025.1625794 (PMC12338046; doi:10.3389/fimmu.2025.1625794)

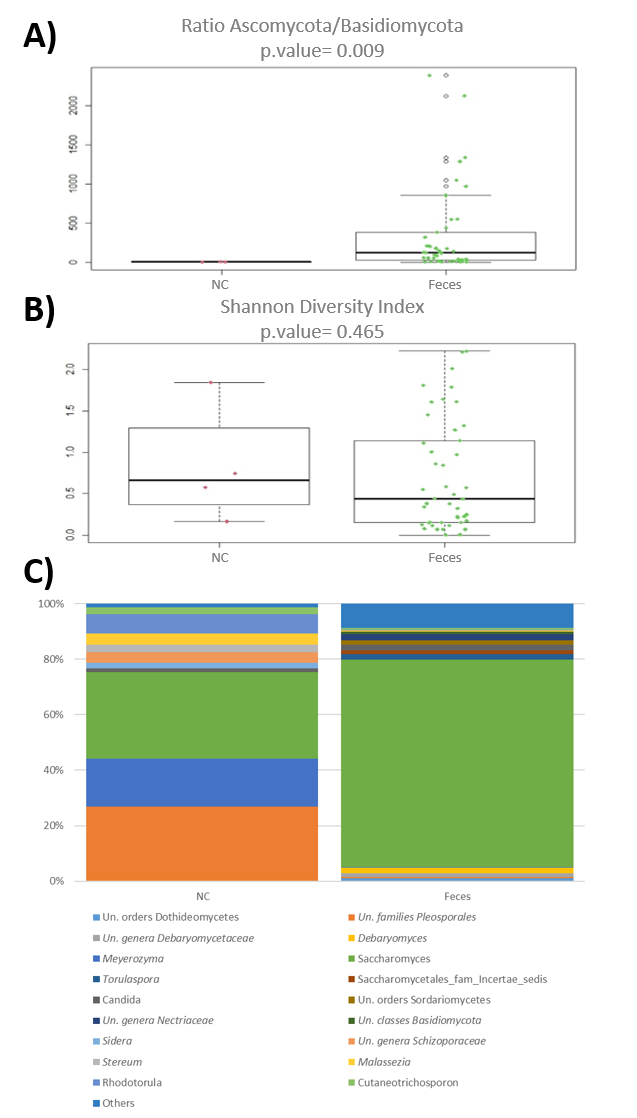

Supplement: Supplementary Figure 1 — Comparison of mycobiome profile in Negative controls (NC) and fecal samples. (A) Boxplot of the Ascomycota/Basidiomycota ratio. (B) Boxplot of the Shannon Diversity index. (C) Barr plot of the 10 more abundant taxa in the samples. Un. orders Dothideomycetes: Unassigned orders in the class Dothideomycetes; Un. genera Debaryomycetaceae: Unassigned genera in Debaryomycetaceae family; Un. genera Nectriaceae: Unassigned genera in Nectriaceae family; Un. families Pleosporales: Unassigned families in the order Pleosporales; Un. orders Sordariomycetes: Unassigned orders in the class Sordariomycetes; Un. classes Basidiomycota: Unassigned classes in the phyla Basidiomycota; Un. genera Schizoporaceae: Unassigned genera in Schizoporaceae family. [file Image1.tif]
